# Prospective Longitudinal ctDNA Workflow Reveals Clinically Actionable Alterations in Ovarian Cancer

**DOI:** 10.1200/PO.18.00343

**Published:** 2019-05-03

**Authors:** Jaana Oikkonen, Kaiyang Zhang, Liina Salminen, Ingrid Schulman, Kari Lavikka, Noora Andersson, Erika Ojanperä, Sakari Hietanen, Seija Grénman, Rainer Lehtonen, Kaisa Huhtinen, Olli Carpén, Johanna Hynninen, Anniina Färkkilä, Sampsa Hautaniemi

**Affiliations:** ^1^ Research Program in Systems Oncology, University of Helsinki, Finland; ^2^ Turku University Hospital, Turku, Finland; ^3^ Institute of Biomedicine, University of Turku, Turku, Finland; ^4^ Helsinki University Hospital, Helsinki, Finland; ^5^ Dana-Farber Cancer Institute, Harvard Medical School, Boston, MA

## Abstract

**PURPOSE:**

Circulating tumor DNA (ctDNA) detection is a minimally invasive technique that offers dynamic molecular snapshots of genomic alterations in cancer. Although ctDNA markers can be used for early detection of cancers or for monitoring treatment efficacy, the value of ctDNA in guiding treatment decisions in solid cancers is controversial. Here, we monitored ctDNA to detect clinically actionable alterations during treatment of high-grade serous ovarian cancer, the most common and aggressive form of epithelial ovarian cancer with a 5-year survival rate of 43%.

**PATIENTS AND METHODS:**

We implemented a clinical ctDNA workflow to detect clinically actionable alterations in more than 500 cancer-related genes. We applied the workflow to a prospective cohort consisting of 78 ctDNA samples from 12 patients with high-grade serous ovarian cancer before, during, and after treatment. These longitudinal data sets were analyzed using our open-access ctDNA-tailored bioinformatics analysis pipeline and in-house Translational Oncology Knowledgebase to detect clinically actionable genomic alterations. The alterations were ranked according to the European Society for Medical Oncology scale for clinical actionability of molecular targets.

**RESULTS:**

Our results show good concordance of mutations and copy number alterations in ctDNA and tumor samples, and alterations associated with clinically available drugs were detected in seven patients (58%). Treatment of one chemoresistant patient was changed on the basis of detection of *ERBB2* amplification, and this ctDNA-guided decision was followed by significant tumor shrinkage and complete normalization of the cancer antigen 125 tumor marker.

**CONCLUSION:**

Our results demonstrate a proof of concept for using ctDNA to guide clinical decisions. Furthermore, our results show that longitudinal ctDNA samples can be used to identify poor-responding patients after first cycles of chemotherapy. We provide what we believe to be the first comprehensive, open-source ctDNA workflow for detecting clinically actionable alterations in solid cancers.

**CONTEXT**

**Key Objective**
The aim of the study was to identify the clinical usefulness of circulating tumor DNA (ctDNA) in the treatment of high-grade serous ovarian cancer (HGSOC) and to provide a clinical workflow for reliable detection of ctDNA alterations.
**Knowledge Generated**
We show that longitudinal ctDNA sampling can be used to detect response to platinum-taxane primary therapy after the first cycles of chemotherapy and to identify clinically applicable mutations and copy number alterations. In one patient with chemoresistant HGSOC, ctDNA-guided treatment including trastuzumab was administered during tumor progression and yielded tumor shrinkage.
**Relevance**
The early detection of poor-responding patients allows the design of alternative treatment strategies in HGSOC. Especially for platinum-resistant patients who have limited treatment options, rapid discovery of resistant cell populations can provide an early opportunity to interfere with the development of recurrence. The provided approach allows the selection of treatment options that target subclones that persist during therapy. This is a substantial improvement in the management of recurrent solid cancers, where tumors are not usually sampled either because of the risk of potential intervention complications or simply because the sample could be insufficient or not representative of the disease.

## INTRODUCTION

Effective treatment of metastatic solid cancers is hampered by intrapatient heterogeneity, tumor evolution, and the paucity of representative tissue samples to guide treatment decisions. Analysis of circulating tumor DNA (ctDNA) is an approach with the potential of overcoming all three obstacles.^1^ Indeed, ctDNA sampling is a clinically attractive, minimally invasive technique that is based on the observation that tumor cells leak DNA to the bloodstream, where it can be captured by genomic assays. ctDNA can be used to monitor tumor evolution,^2^ detect cancer early,^3^ and monitor treatment efficacy.^4^ However, the value of ctDNA in guiding treatment decisions still remains controversial,^1^ especially in cancers lacking dominant oncogenic mutations.

A major reason for controversy in the use of ctDNA to guide clinical decision making stems from the varying, and often poorly described, analysis approaches.^5^ Translating genomic alterations in ctDNA data into a clinical decision requires multidisciplinary expertise in medical genetics, bioinformatics, and clinical oncology, as discussed in response to a recent study.^6^ Another reason is that most earlier studies focused on a few or tens of candidate genes, which significantly decreases the probability of finding an effective therapy on the basis of ctDNA data, whereas exome or whole-genome ctDNA sequencing is currently too expensive for clinical use.

Here, we present a comprehensive ctDNA analysis approach that consists of longitudinal ctDNA sampling, a panel of more than 500 cancer-related genes, ctDNA analysis–tailored bioinformatics pipelines, and a Translational Oncology Knowledgebase to identify clinically actionable mutations and copy number alterations (CNAs). We demonstrate the usefulness of this approach in high-grade serous ovarian cancer (HGSOC), which is the most common and aggressive form of epithelial ovarian cancer, with a 5-year survival rate of 43%.^7^ The current standard-of-care therapy for advanced HGSOC consists of cytoreductive surgery and platinum-paclitaxel chemotherapy.^8^ Although the initial response to standard-of-care therapy is usually good, nearly all patients eventually relapse and have limited treatment options. Thus, there is an urgent need to find effective therapy targets for HGSOC in general and for relapsed, chemotherapy-resistant disease in particular.

## PATIENTS AND METHODS

### Patients

We prospectively collected 78 plasma samples from 12 patients treated for ovarian or primary peritoneal HGSOC at Turku University Hospital, Finland (Table 1 and Data Supplement). Patients were treated either with primary debulking surgery (PDS) followed by a median of six cycles (range, five to six cycles) of platinum-taxane chemotherapy, or with neoadjuvant chemotherapy (NACT), where the primary operation was laparoscopic evaluation with diagnostic tumor sampling, followed by three cycles of carboplatin and paclitaxel (Data Supplement). In these patients, an interval debulking surgery (IDS) was performed after NACT, aimed at complete cytoreduction, followed by a median of three cycles (range, three to six cycles) of adjuvant chemotherapy.

**TABLE 1.**
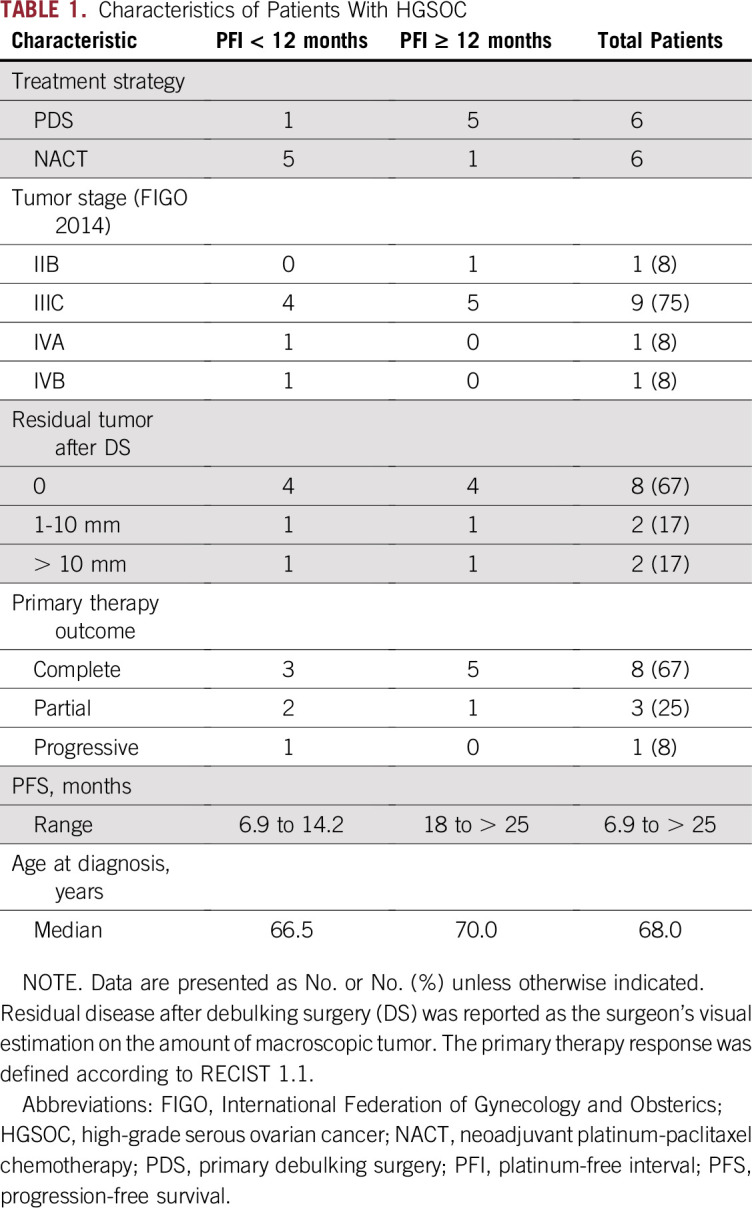
Characteristics of Patients With HGSOC

Patients were stratified into poor and good responders on the basis of the platinum-free interval (PFI).^9^ Patients with platinum-resistant (PFI, less than 6 months; n = 3) or partially platinum-sensitive (PFI, 6 to 12 months; n = 3) disease were regarded as poor responders (50%), whereas patients with platinum-sensitive disease (PFI, greater than 12; n = 6) were considered good responders (Table 1 and Data Supplement).

All patients participating in the study gave their informed consent, and the study was approved by the Ethics Committee of the Hospital District of Southwest Finland.

### Sample Preparation and Sequencing

Venous whole blood (5 to 6 mL) was collected into an EDTA tube, shaken eight to 10 times, and centrifuged twice for 10 minutes at 2000 × *g*. The samples were stored at −80°C as 1 to 2 mL aliquots frozen less than 2 hours after collection to avoid lysis of the white blood cells.^1^ Cell-free DNA (cfDNA) was extracted from plasma samples and subjected to 1000× targeted Illumina Hi-Seq sequencing at BGI (BGI Europe A/S, Copenhagen, Denmark) using the Oseq Solid Cancer Panel with more than 500 clinically actionable genes (Data Supplement).

DNA was isolated from 21 liquid LN_2_ snap-frozen tumor tissue and ascites samples (Data Supplement), as well as from whole blood (germline) buffy coat samples, and was sequenced using the Oseq panel with coverage of 200×.

### Mutation and Copy Number Detection

Mutation detection was performed in the computational ecosystem Anduril^10^ using the ctDNA data analysis pipeline, which is based on best practices for sequencing data analysis (Data Supplement). A detailed description of the pipeline and validation data can be found in the Data Supplement. Briefly, somatic single nucleotide variants (SNVs) and indels were detected using Mutect2.^11^ We tracked mutations detected in at least one plasma or tumor tissue sample with at least 1% variant allele frequency (VAF) but not in the corresponding germline blood control (maximum two reads or VAF 0.001 accepted). Filtering steps included detection of likely spurious mutations by identifying clusters of variants shared between patients but not known in cancer,^12^ and functionally irrelevant variants by a Combined Annotation Dependent Depletion^13^ score of less than 15. CNAs were estimated using PanelDoC^14^ (Data Supplement) and CNVPanelizer.

Detected somatic alterations were used to confirm the presence and quantity of ctDNA (total burden). The proportion of ctDNA in cfDNA was evaluated from *TP53* mutation^4^ VAF identified as truncal mutations from the pretreatment tissue (VAF range, 1.1% to 97.6%) and plasma samples (VAF range, 0.8% to 58.1%; Data Supplement). To estimate the change in mutational profiles, we identified the largest proportion of significantly decreased variants during treatment compared with the pretreatment sample (*P* < .01, Fisher’s exact test). Because of varying numbers of mutations in the pretreatment samples, the proportion of significantly decreased variants was corrected for mutation counts.

Pathway analysis was performed for genes with a nondecreasing mutation frequency (*P* < .01, Fisher’s exact test; Data Supplement) using the ConsensusPathDB^15^ algorithm. The ConsensusPathDB integrates data from 32 databases and allows comprehensive over-representation analysis for mutations.

### Immunohistochemistry and In Situ Hybridization

Potentially clinically actionable alterations were validated through immunohistochemistry (IHC) and in situ hybridization for alterations classified as most prominent (ESCAT, the European Society for Medical Oncology Scale for Clinical Actionability of Molecular Targets)^16^ and shown to exist in patients’ tumor tissue (Data Supplement).

## RESULTS

### ctDNA Panel Reliably Captures Mutations and CNAs

**FIG 1.**
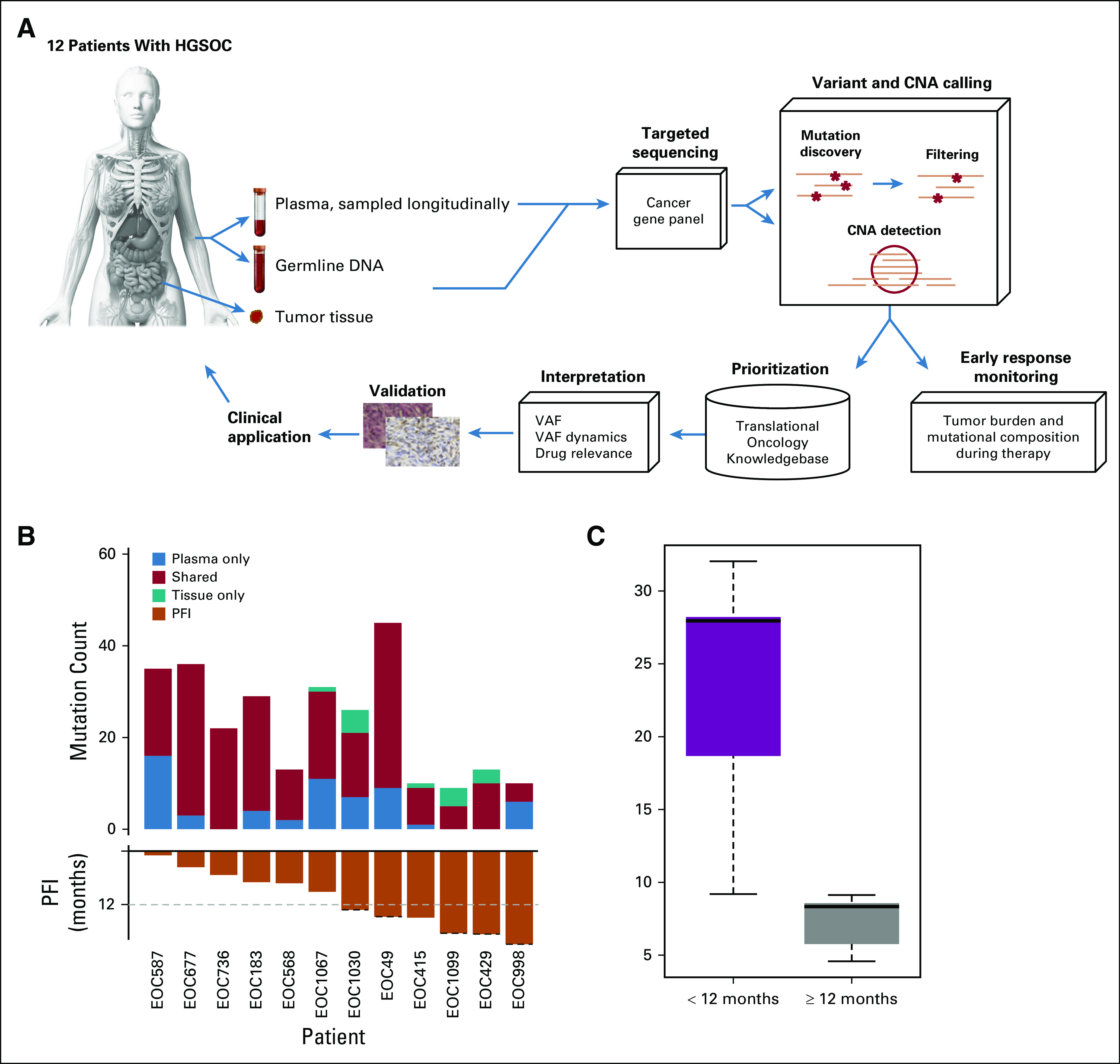
Comprehensive circulating tumor DNA (ctDNA) analysis approach identifies mutations and copy number alterations (CNAs) in high-grade serous ovarian cancer (HGSOC). (A) Analysis pipeline to detect genomic alterations from longitudinal ctDNA sampling of patients with HGSOC. After data analysis, the alterations are used to monitor patient responses and are prioritized on the basis of evidence for alterations and existing therapies. (B) Concordance between mutations detected from plasma samples and tumor tissue samples from the same patient. (C) During the treatment, a lower ratio of mutations with decreasing variant allele frequencies (VAFs) and larger mutation counts were detected in the poor-responding patients compared with the good-responding patients (*P* = .008). Good-responding patients showed either a small number of mutations or a large proportion of decreasing mutations during treatment. PFI, platinum-free interval.

We collected 78 longitudinal ctDNA samples and 21 tumor tissue samples from 12 unselected patients with HGSOC (Data Supplement). We applied a sequencing panel of more than 500 genes, followed by variant and CNA calling, filtering, and prioritization of clinically relevant aberrations as shown in Fig 1A and the Data Supplement. The motivation for ctDNA analysis was to perform a minimally invasive genomic survey of clinically actionable tumor aberrations in the patient. To establish this connection, we used tumor tissue samples as a ground truth for patient mutational burden. Altogether, 265 mutations in 185 genes (Data Supplement) and CNA aberrations in 113 genes (Data Supplement) passed our calling and filtering criteria.

**FIG 2.**
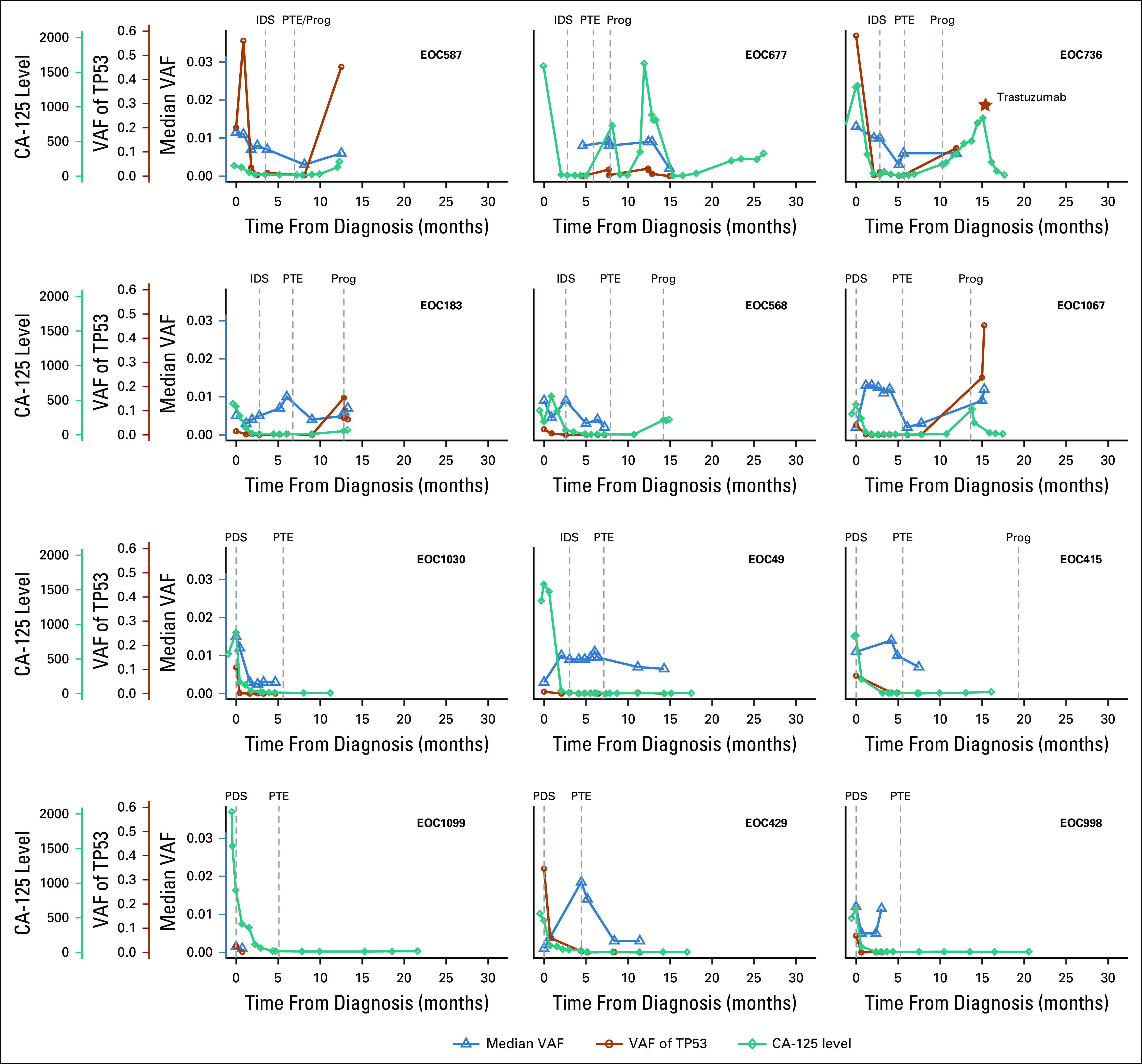
Tumor burden measured by circulating tumor DNA (ctDNA), *TP53*, and cancer antigen 125 (CA-125) during therapy and follow-up. Tumor burden was estimated from ctDNA through *TP53* variant allele frequency (VAF) (orange lines) and median VAF (blue lines). Correlation between tumor content and CA-125 was 0.67. The start of the trastuzumab treatment during progression (Prog) in patient EOC736 has been marked with a star. More detailed figures, including all treatments administered to each patient, are given in the Data Supplement. IDS, interval debulking surgery; PDS, primary debulking surgery; PTE, primary treatment end.

The median concordance of mutations detected from plasma to tumor tissue samples from the same patient was 79% (Fig 1B; Data Supplement). Similar median concordance (74%) was observed with CNAs for high tumor content (greater than 30%) plasma samples (Data Supplement). Mutations in the *TP53* gene are reported in nearly 100% of patients with HGSOC.^4^ In our cohort, *TP53* mutations were observed in all patients. We noted, consistent with previous studies,^4^ a moderate positive correlation between ctDNA *TP53* VAF and serum cancer antigen 125 (CA-125; IU/mL) levels (median, 0.67; range, 0.16 to 0.97; Fig 2; Data Supplement). The number of detected CNAs highly correlated with the proportion of ctDNA from cfDNA (n = 101; correlation coefficient, 0.88; Data Supplement), consistent with earlier studies.^17^ These results show that the mutations and CNAs detected from plasma are in good concordance with those detected from tissue samples, which is the prerequisite for the use of detected mutations and CNAs from ctDNA in clinical decision making.

### CtDNA Mutation Profiles Reflect Long-Term Treatment Response and Reveal Targetable Pathways in Patients With Aggressive HGSOC

ctDNA allows several ways to monitor treatment response. For HGSOC, Parkinson et al^4^ suggested the use of *TP53* measured from ctDNA in disease monitoring. We observed that, consistent with their results, *TP53* VAF was below 0.5% in all except one patient during primary chemotherapy and was increased in all patients at disease progression (Fig 2). Although the mutation profiles within a patient were relatively stable (Data Supplement), the good-responding patients showed significantly fewer mutations and a higher proportion of mutations with decreasing VAF when compared with the poor-responding patients (*P* = .008; Fig 1C). Importantly, changes in the ctDNA mutation frequencies were generally observed early, typically during the first two cycles of chemotherapy (Data Supplement). These results suggest that the changes in VAFs detected from ctDNA samples can be used for the early identification of patients with poor response to chemotherapy.

We hypothesized that genes whose mutation VAFs remained stable or increased during treatment in poor responders could reveal pathways crucial for chemoresistance. Pathways enriched in the poor-responding patients in comparison with the good-responding patients included transcription, p53, and chromatin regulatory pathways and DNA double-strand break repair pathways (Data Supplement). The most selectively enriched pathways in poor-responding patients were related to chromatin regulation and included 10 genes. *KMT2A*, *KMT2B*, *KMT2D*, *KDM5A*, and *SUZ12* encode regulators of H3K4 histone methylation, which is reported to contribute to DNA replication fork degradation and therapy resistance.^18^ *SMARCD1* and *SMARCA4* encode SWI/SNF complex subunits.^19^ The pathways and herein detected mutations provide a basis for finding targets for overcoming treatment resistance.

### CtDNA Identifies Actionable Mutations in Patients With HGSOC

We prioritized and interpreted all 265 mutations and 113 CNAs using the Translational Oncology Knowledgebase, which integrates information from 11 databases and 14 scientific articles (Data Supplement). We identified high-confidence, potentially clinically actionable mutations or CNAs in seven patients (58%; Table 2). The Knowledgebase-prioritized mutations and CNAs revealed four major targetable processes: mammalian target of rapamycin (mTOR; patients EOC429, EOC49), DNA repair (patients EOC429, EOC677, and EOC1067), epidermal growth factor receptor (EGFR; patients EOC587, EOC736, and EOC568), and cyclin dependent kinases (CDKs; patient EOC1067).

**TABLE 2.**
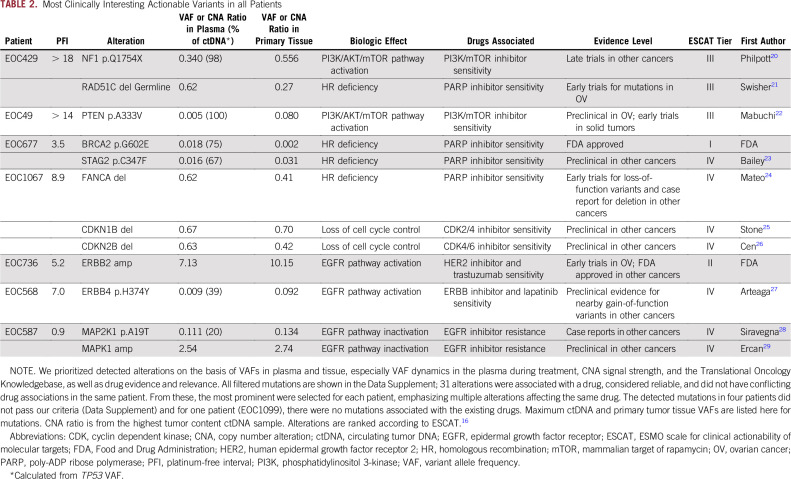
Most Clinically Interesting Actionable Variants in all Patients

**FIG 3.**
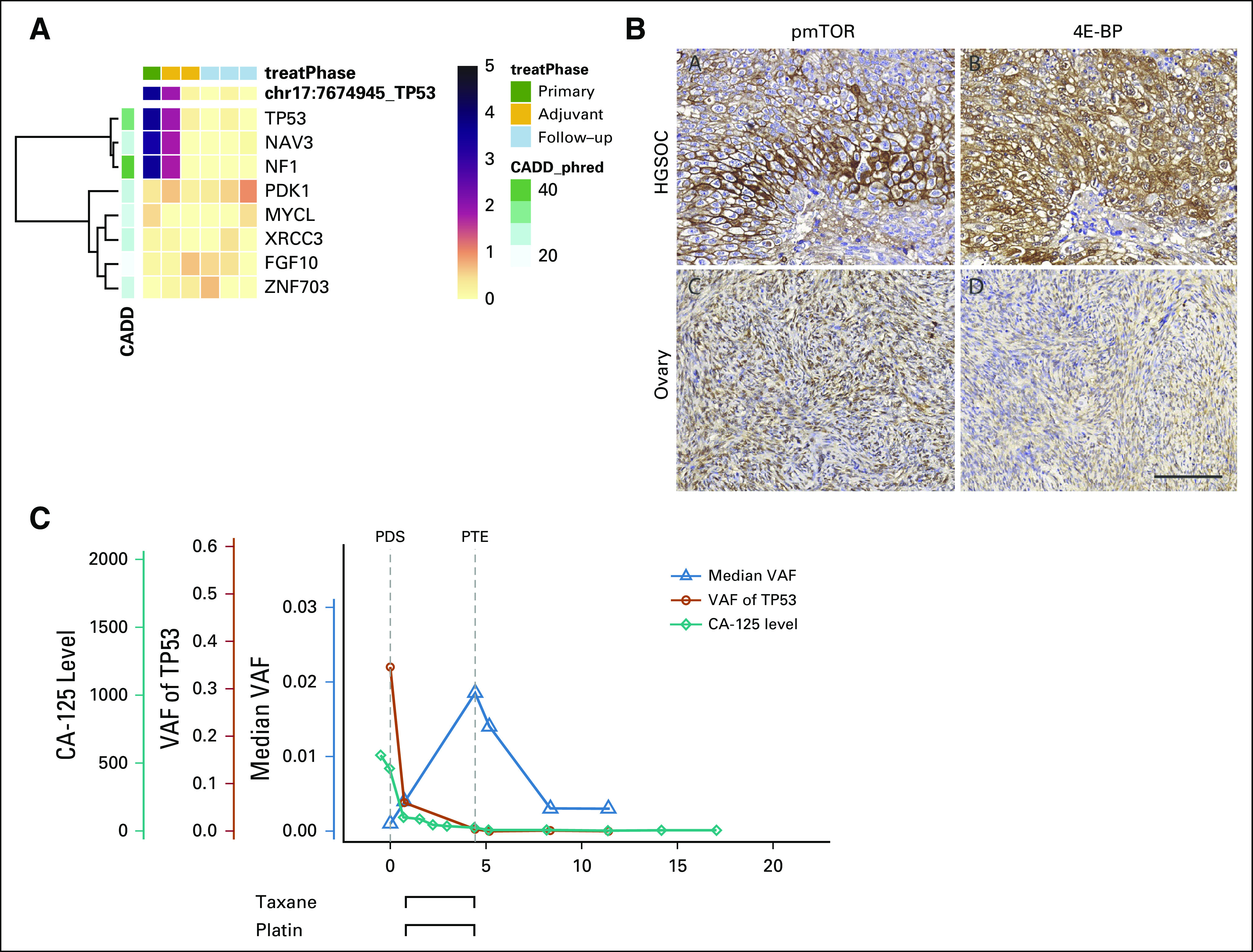
Pathogenic mutation in *NF1* was detected in circulating tumor DNA (ctDNA), leading to functionally overactive mammalian target of rapamycin (mTOR) signaling in the tumor tissue. (A) Mutational frequencies in a good-responding patient (EOC429) during primary treatment and follow-up. Variant allele frequency (VAF) values are normalized for the frequency level to show relative changes in the levels. Mutational profile shows a low mutation rate because only eight mutations were detected. After the last chemotherapy cycle, *TP53* VAF declined to 0.4% from the pretreatment value of 34.6%. No novel mutations were detected during or after the treatment. The *NF1* mutation (Q1775) was detected in samples before and after primary debulking surgery (PDS) with high VAF (34% and 5.4%, respectively). The samples taken during therapy had too low a tumor burden for reliable mutation detection, and *NF1* was not detected in these subsequent samples. (B) mTOR pathway activation was detected in primary tumor tissue with higher staining for phosphorylated mTOR (pmTOR) and E4-BP1 compared with normal ovarian tissue. Scale bar, 100 µm. (C) In addition to having a complete response on the basis of RECIST 1.1, the response to primary therapy was good, as indicated by cancer antigen 125 (CA-125) values that stayed low during treatment and follow-up. A similar pattern was detected for *TP53* VAF. Median VAF shows the prolonged effect of chemotherapy. CADD, Combined Annotation Dependent Depletion; HGSOC, high-grade serous ovarian cancer; PTE, primary treatment end; 4E-BP, 4E-binding protein.

The highest allele frequency mutations matching tumor content were detected in *PTEN* (patient EOC49) and *NF1* (patient EOC429*)*, which cause overactivation of the mTOR pathway.^20,22^ Patients with the activated mTOR pathway have been shown to be responsive to phosphatidylinositol 3-kinase/mTOR inhibitors.^22^ We validated overactivity of the mTOR pathway in the two patients with IHC from tumor tissue samples (Fig 3; Data Supplement). For patient EOC429, we also detected a *PDK1* mutation of unknown function and a *PIK3CA* amplification, which potentially cause phosphatidylinositol 3-kinase/mTOR pathway activation. Both patients with the mTOR pathway alterations responded well to platinum-based therapy (Fig 2). In patient EOC49, *PTEN* mutation VAF in omentum (8% and 6% for pretreatment and interval samples, respectively) and plasma matched the pattern of *TP53* mutation, indicating that the *PTEN* mutation exists in all tumor cells and presents an early event in tumor evolution (Data Supplement). *PTEN* was detected in a follow-up sample at 8 months after primary treatment with VAF of 0.1%, confirming its persistence during therapy.

We observed mutations and CNAs in genes associated with DNA repair in three patients. In patient EOC677, we detected mutations in *BRCA2* and *STAG2*, which potentially confer homologous recombination (HR) DNA repair deficiency and sensitivity to poly-ADP ribose polymerase (PARP) inhibitors. However, the frequencies of these mutations were low in the tumor tissues (0.2% for *BRCA2* and 3.1% for *STAG2*), suggesting subclonal events. In patient EOC429, we detected a germline deletion in *RAD51C* from plasma, which has been shown to confer HR deficiency and PARP inhibitor sensitivity,^21^ and, consistent with this, the patient had a prolonged response to platinum-based chemotherapy. We also detected a somatic deletion of *FANCA* in patient EOC1067. This patient also had concurrent deletions in *CDKN1B* and *CDKN2B*. These alterations are predicted to cause sensitivity to CDK2/4^25^ and/or CDK4/6 inhibitors.^26^ Regardless of the presence of HR-deficiency–conferring alterations, this patient, who had no residual tumor after surgery, progressed 8.9 months after the last platinum cycle.

**FIG 4.**
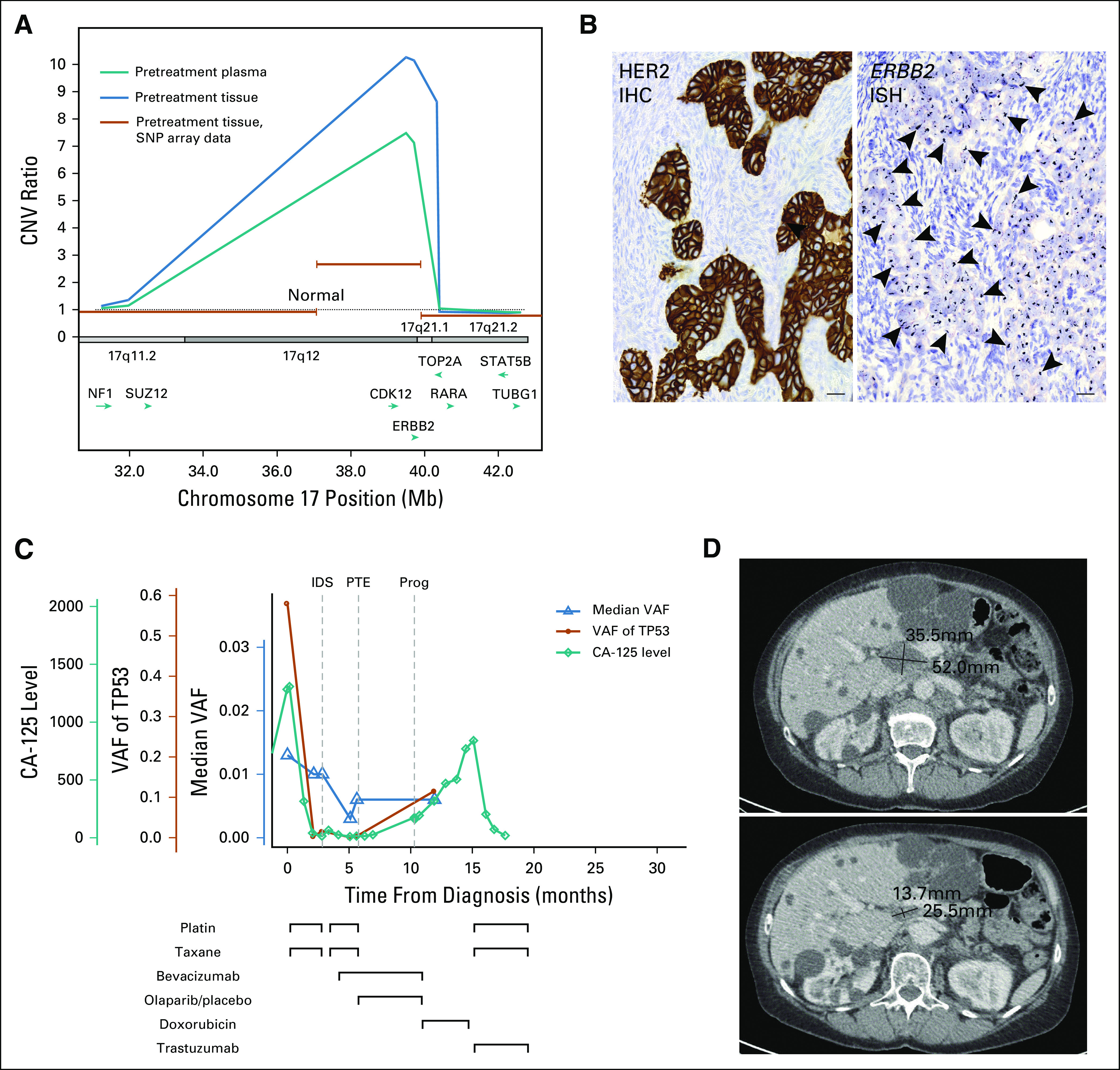
*ERBB2* amplification and the effect of combination treatment with trastuzumab, platinum, and paclitaxel for patient EOC736. (A) *ERBB2* amplification was detected from a primary plasma sample and adnexal cancer tissue removed at primary debulking surgery. The amplified region was verified from primary omental metastatic tissue using SNP-array data (orange lines). Amplified region contains multiple genes, of which *CDK12* and *ERBB2* were included in the targeted panel. *RARA* was within the amplified region in plasma but not in primary omentum. (B) ERBB2 protein was overexpressed in cancer tissue (arrowheads) in immunohistochemistry (IHC) and the *ERBB2* gene was amplified in DNA in situ hybridization (ISH) analysis (scale bars, 20 µm). (C) Treatments and responses measured by circulating tumor DNA (ctDNA) and cancer antigen 125 (CA-125). Trastuzumab and reduced-dose platinum-paclitaxel treatment led to normalization of CA-125 values, from 800 to 19, after only three treatment cycles. (D) The pretreatment computed tomography scan (upper part) shows the largest diameters of the relapsed tumor. After 14 weeks (four cycles) of combination chemotherapy, there was an average 52% reduction in the diameters of the lesion (lower part). The patient had multiple apparently benign cysts in the liver and the right kidney that were unrelated to ovarian cancer. CNV, copy-number variation; HER2, human epidermal growth factor receptor 2; IDS, interval debulking surgery; Prog, progression; PTE, primary treatment end; SNP, single-nucleotide polymorphism; VAF, variant allele frequency.

Three patients had mutations or CNAs associated with sensitivity to drugs targeting the epidermal growth factor receptor (EGFR) pathway. Patient EOC568 had an *ERBB4* mutation, which is predicted to confer sensitivity to EGFR inhibitors.^27^ Patient EOC587 had a *MAPK* mutation and a simultaneous *MAPK1* amplification, which are associated with resistance to the EGFR targeting therapies.^28,29^ We detected a high copy-count *ERBB2* amplification in the ctDNA in patient EOC736. The *ERBB2* locus is amplified in 3% to 11% of HGSOCs^30,31^ and is a predictive biomarker for trastuzumab. The *ERBB2* amplification was validated in primary omentum tissue using SNP genotype–based CNA detection and in the tumor tissue sample from interval debulking surgery with in situ hybridization and IHC (Figs 4A and 4B).

Patient EOC736 was treated with NACT, and she participated in a clinical trial after primary treatment, receiving maintenance bevacizumab and olaparib or placebo (Fig 4C). Although she had a clinical and radiologic complete response to primary therapy, *TP53* VAF remained detectable throughout the treatment, which, on the basis of our findings, indicates a poor response to platinum taxane (Data Supplement). Indeed, disease progression was detected only 5 months after the end of primary therapy. This patient also suffered from severe paclitaxel-induced neuropathy after primary treatment, and therefore, pegylated liposomal doxorubicin (PLD) was started at the first relapse instead of dose-dense paclitaxel therapy. PLD was discontinued after four cycles because of constantly rising CA-125 (from 193 to 769) and progression in lymph nodes as evidenced by a computed tomography scan.

On the basis of the *ERBB2* amplification detected in ctDNA, treatment of patient EOC736 was changed to trastuzumab 600 mg subcutaneously once every 3 weeks, combined with reduced doses of carboplatin (AUC4) and dose-dense paclitaxel (80 mg/m^2^ on days 1 and 8). The patient responded to this combination well, and a biochemical complete response (CA-125 value reduced from 840 IU/mL to 19 IU/mL) was achieved after only three treatment cycles (Fig 4C). The response-evaluation computed tomography scan showed a notable reduction in the size of a mesenteric lesion, and partial response was achieved according to the Response Evaluation Criteria in Solid Tumors (RECIST) 1.1 criteria (Fig 4D).

## DISCUSSION

The value of ctDNA has been demonstrated in early detection and disease monitoring, but evidence for using ctDNA to guide clinical decision making is weaker.^1^ Herein, we present a clinical workflow that combines targeted sequencing of more than 500 genes from liquid biopsies, a ctDNA data analysis pipeline, and a Translational Oncology Knowledgebase to enable longitudinal analysis of ctDNA during therapy and guide personalized cancer treatment decisions. This approach allows for reliable quantification of somatic mutations and CNAs, as well as improved detection of subclonal mutation clusters over the course of disease. The ctDNA analysis pipeline and Translational Oncology Knowledgebase are open-source and available with documentation (Data Supplement).

We detected high ctDNA–tumor congruence, which, together with the absence of alterations from the blood cells used as germline samples and comprehensive filtering of mutations through clustering, maximizes the likelihood that the reported alterations originated from tumor and not from other sources, such as clonal hematopoiesis.^1^ Indeed, our filtering approach using longitudinal samples removed false-positive alterations in all patients. Our results further emphasize the possibility that tissue samples may not represent the progressive tumor in all cases. This highlights the potential of ctDNA as a clinically feasible, noninvasive method to uncover genetic alterations and drug targets during disease progression. Thus, the optimal discovery of clinically useful alterations requires multiple samples from one patient combined with careful cross-disciplinary interpretation conducted by geneticists, bioinformaticians, and clinicians.

Longitudinal ctDNA sampling can be used to monitor response to first-line therapy and to identify poor-responding patients in HGSOC.^4^ The early hints of chemoresistance (ie, lower ratio of mutations whose frequency is reduced and smaller decrease of tumor burden), may be used to predict prognosis and open a window of opportunity for therapeutic interventions before disease relapse and additional evolution of the tumor. The most informative comparisons for predicting treatment outcome are samples at the preoperative stage, after two cycles of chemotherapy, and after debulking surgery, because they provide information on treatment response and on persisting tumor subclones

We identified potentially clinically applicable mutations or CNAs in more than one half of the patients with HGSOC. These included two patients with mTOR pathway–activating mutations that were validated. Both patients had a good response to the platinum-based first-line therapy, but in the case of relapse they could most likely benefit from mTOR inhibitors alone or in combination with chemotherapy.^22^ In patient EOC429, we identified alterations conferring both mTOR pathway activation and HR deficiency, which suggests potential benefit from combination treatment with, for example, mTOR inhibition and PARP inhibition. In addition, two patients had more than one concurrent, potentially targetable alteration. Patient EOC1067 progressed after 9 months of platinum-based therapy despite the *FANCA* deletion conferring HR deficiency, potentially because of the contribution of the CDK alterations on cell-cycle regulation and tumor progression.^32^ On the basis of the concurrent *CKDN1B* and *CKDN2B* deletions, this patient could potentially benefit from a combination targeting both pathways, for instance, PARP inhibitor and CDK4/6 inhibitor. These results indicate that longitudinal ctDNA sampling can detect and monitor a relapse at an early stage and identify potentially effective drug combinations.

We identified a high-confidence *ERBB2* amplification in the ctDNA of a patient with platinum-resistant HGSOC and used this information to change the patient’s treatment to include trastuzumab with reduced doses of platinum and paclitaxel. This patient had relapsed 5 months after primary platinum-taxane–based therapy and did not respond to PLD. Thus, rapid response to the ctDNA-guided treatment, detected by complete normalization of CA-125 values within 2 months after starting the combination treatment, was surprising. Earlier studies that have combined ERBB2 inhibitors with platinum and paclitaxel report similar encouraging responses for relapsed patients with advanced HGSOC.^31^ Although the number of patients in these studies and our study remains small because of the low number of patients with *ERBB2*-amplified HGSOC, these results warrant testing *ERBB2* amplification from relapsed HGSOC patients with advanced disease.

ctDNA sampling and sequencing with a large panel offers several benefits to physicians. Response to therapy can be inferred using two or three consecutive ctDNA samples during therapy. Rapid discovery of resistant cell population expansion provides an early opportunity to interfere with the development of recurrence. This is particularly important in chemoresistant relapse in which larger tumor burden is associated with low therapy responses, with both conventional and targeted therapies. Moreover, large gene panels offer a higher probability of identifying clinically relevant mutations and CNAs, as compared with targeted approaches with a few targets. This is a substantial improvement in managing recurrent solid cancers in which tumors are not usually sampled either because of the risk of potential intervention complications or because the sample could be insufficient or not representative of the disease.
